# The Small G Protein AtRAN1 Regulates Vegetative Growth and Stress Tolerance in *Arabidopsis thaliana*

**DOI:** 10.1371/journal.pone.0154787

**Published:** 2016-06-03

**Authors:** Peipei Xu, Aiping Zang, Haiying Chen, Weiming Cai

**Affiliations:** Institute of Plant Physiology and Ecology, Shanghai Institutes for Biological Sciences, Chinese Academy of Sciences, 300 Fenglin Rd, Shanghai 200032, China; National Taiwan University, TAIWAN

## Abstract

The evolutionarily conserved small G-protein Ran plays important role in nuclear translocation of proteins, cell cycle regulation, and nuclear envelope maintenance in mammalian cells and yeast. *Arabidopsis* Ran proteins are encoded by a family of four genes and are highly conserved at the protein level. However, their biological functions are poorly understood. We report here that *AtRAN1* plays an important role in vegetative growth and the molecular improvement of stress tolerance in *Arabidopsis*. *AtRAN1* overexpression promoted vegetative growth and enhanced abiotic tolerance, while the *atran1 atran3* double mutant showed higher freezing sensitivity than WT. The *AtRAN1* gene is ubiquitously expressed in plants, and the expression levels are higher in the buds, flowers and siliques. Subcellular localization results showed that *AtRAN1* is mainly localized in the nucleus, with some present in the cytoplasm. *AtRAN1* could maintain cell division and cell cycle progression and promote the formation of an intact nuclear envelope, especially under freezing conditions.

## Introduction

The small soluble GTP-binding protein Ran has been shown to be important for the nuclear translocation of proteins and cell cycle regulation in mammalian and yeast cells [1–3]. Ran is mainly localized in the nucleus. Ran exerts its biological function by binding and hydrolyzing GTP. However, intrinsic nucleotide exchange and GTP hydrolysis involving Ran are very low [4–5]. Many types of regulatory proteins have been identified that interact specifically with Ran and stimulate its nucleotide exchange and GTP hydrolysis rates for thousands of times [6]. The identification of RanBP1 (Ran binding protein) and RanGAP (Ran GTPase-activating protein) made the function of Ran more clear in plants [5, 7]. RanGAP and its co-activator RanBP are excluded from the nucleus and deplete RanGTP from the cytoplasm.

Four Ran GTPases (*AtRAN1*, *AtRAN2*, *AtRAN3*, *AtRAN4*) have been identified in the *Arabidopsis* genome by comparing amino acid similarities [8–9]. Ran GTPases interact directly with AtKPNB1 (a homolog of human KPNB1), which is involved in abscisic acid response and drought tolerance [10]. MOS14 (modifier of snc1-1, 14) interacts with AtRAN1 and is required for the proper splicing of resistance genes and plant immunity [11]. *RAN1* mediates seed development through its parental ratio by affecting the onset of endosperm cellularization [12]. The interaction of AtRAN2 with PHIP1 (Phragmoplastin interacting protein 1) may play a unique role in mRNA polarization [13]. With the aid of AtRAN3, AtMBD5 becomes localized to the vicinity of chromosomes during cell division and may play an important role in the maintenance of chromatin structures [14]. Wheat *RAN1* is involved in the regulation of cell division and alters the primordial meristem, mitotic progression, and sensitivity to auxin in rice and *Arabidopsis* [15]. Decreased ATP levels induced by oxidative stress lead to decreased Ran-GTP levels and disordered Ran distribution [16]. In rice, the expression of *OsRAN1* is induced by jasmonic acid [17]. The overexpression of *OsRAN2* affects the sensitivity to salt stress in rice [18]. *OsRAN1* and *OsRAN2* play important roles in cold tolerance [19–20]. In this report we indicate that *AtRAN1* not only involved in vegetative growth and seed yield but also regulates stress tolerance partly redundant with *AtRAN1*. *AtRAN1* could promote cell cycle progression and maintain nuclear envelop under freezing stress. The sweet potato Ran genes show differential transcriptional regulation in response to various environmental stresses with tissue specificity [21]. *Lepidium latifolium* L. (LlaRan) is involved in cold stress [22]. Therefore, RAN proteins not only have a major impact on plant development but also mediate plant responses to the environment.

Abiotic stresses often affect plant growth and crop productivity, resulting in great reductions in output [23]. The identification of stress response genes is very important to plant survival and yields under stress conditions [24]. It is well known that nucleo-cytoplasmic trafficking of proteins can affect post-transcriptional regulation, such as mRNA processing, RNA import/export to/from the nucleus, and other processes. [25]. The nucleo-cytoplasmic trafficking of proteins through Ran-dependent pathways may play an important role in the response to abiotic stress [26]. For example, *AtKNB1*, an Arabidopsis homolog gene of human importin β1, is required for ABA response and drought tolerance [27]. *SAD2* (super sensitive to ABA and drought 2) encodes an importin beta-domain family protein likely to be involved in nuclear transport in ABA signaling and UV stress [28].

Normally, plants in temperate regions can increase their freezing tolerance through exposure to chilling temperatures in a process known as cold acclimation [29–30]. This process can result in greatly altered gene expression, biomembrane lipid composition, and other qualities [31]. Unpredictable cold snaps can result in pollen sterility at the flowering stage [32]. The maintenance of cell division, especially under cold stress, is important to plant survival and growth. In this study, we explored the roles of *AtRAN1* in the cell cycle progression and regulation of stress tolerance in *Arabidopsis*.

Plant growth is an important yet poorly understood biological process [33–34]. The investigation of the *AtRAN1* gene is therefore helpful for our comprehension of plant growth mechanisms. By using *Arabidopsis AtRAN1-*knockdown and *AtRAN1-*overexpressing lines, we demonstrate that *AtRAN1* controls plant growth, stress resistance and yield. Our results imply that plant Ran GTPase plays an important role in the link between environmental cues and growth processes in plants.

## Materials and Methods

### Plant material and growth conditions

*Arabidopsis* (*Arabidopsis thaliana* L., Heynh. ecotype Columbia (Col-0)) was used in this study. The T-DNA mutants *atran1-1* (SALK_138680), *atran1-2* (SALK_067649), *atran2* (SALK_123620C) and *atran3* (SALK_074683) were obtained from the Arabidopsis Biological Resource Center (http://abrc.osu.edu/) and genotyped by polymerase chain reaction (PCR) using primers flanking the insertions (S3 Table). To generate the double mutant, we crossed *atran3* with *atran1-1* and *atran1-2*, *atran3* with *atran2*, and the genotypes of the F2 plants were identified by PCR. The *Arabidopsis* seeds were surface-sterilized and planted in MS medium (Murashige & Skoog 1962) containing 1% sucrose. Plates were maintained in the dark at 4°C for 2 d to synchronize germination and then transferred to a growth chamber under a 16/8 h photoperiod. The flowering time was determined by counting the days and rosette leaves once a flower bud was visible.

### Plasmid constructions and generation of transgenic plants

The full-length cDNA of *AtRAN1* and *AtRAN3* was amplified from Col-0 wild-type cDNA. The modified green fluorescent protein (GFP) gene was amplified using the pCAMBIA1302 vector. To construct the plasmid for gene overexpression, *AtRAN1* and *AtRAN1*::*GFP* were cloned into a pHB vector [35] to generate 35S::*AtRAN1* and *35S*::*AtRAN1*::*GFP* transgenic lines. All reagents and enzymes used here for PCR amplification or restriction digestion were purchased from Takara, Japan. *Arabidopsis* plants (ecotype Columbia) were transformed by the floral-dipping method [36]. Hygromycin (Roche) resistance was used to screen positive transgenic plants. The concentration of hygromycin used for screening the plants was 50 mg/L. Genomic PCR was used to confirm transgenic plants with primers specific for the hygromycin phosphotransferase (HPT) gene. Semi-quantitative reverse transcription (RT)-PCR and quantitative PCR (qPCR) were performed to detect the gene expression levels in transgenic *Arabidopsis*. The primers used are shown in Supplementary S1 and S2 Tables at Plos one online.

### Subcellular localization study

The GFP signal was observed in the roots of 1-week-old transgenic *Arabidopsis* seedlings, which were constitutively transformed with constructs of double *35S*::*AtRAN1*::GFP and analyzed by using confocal microscopy (Zeiss LSM510; Jena, Germany). In addition, transient expression of the double *35S*::*AtRAN1*::GFP construct was observed in tobacco epidermal cells.

### Freezing, Salt and ABA treatment assay

Freezing treatment was performed as previously described [37]. The *atran1-1*, *atran1-2*, *atran2*, *atran3*, *atran1-1 atran3*, *atran1-2 atran3*, *AtRAN1-OE1*, *AtRAN1-OE3* and wild-type plants were grown in a growth chamber under 16-h-light (23°C)/8-h-dark (21°C) cycles until treatment. For freezing treatment, the seedlings were placed in a controlled temperature chamber and subjected to freezing stress. Soil-grown plants (4 weeks old) or 10-d-old seedlings grown on MS medium plates were subjected to a temperature drop from 4°C to -2°C in the growth chamber. When the temperature reached -2°C, ice crystals were placed on the plates or soil to induce crystallization. After one hour at -2°C, the temperature was lowered to the final temperature. After treatment at the final temperature, the plants or seedlings were thawed at 4°C overnight. Following recovery for 14 d under 16-h-light (23°C)/8-h-dark (21°C) cycles, the plants were photographed. Salt treatment was performed using seedlings in MS medium containing 100 mM NaCl. ABA treatment was performed using seedlings in MS medium containing 0.5 μM ABA. The plants were photographed after treatment.

### Ion leakage assay

The electrolyte leakage test was performed as previously described [38]. The electrolyte leakage assay is a widely used means to assess the extent of plant injury in relation to temperature stress. The test is based on the principle that damage to cell membranes results in enhanced leakage of solutes into the apoplastic water [39].

### RT-PCR and real-time PCR

The synthesis of cDNA was performed using the ReverTra Ace qPCR Master Kit (FSQ-201) as previously described [40–42]. An aliquot of 2 μL of ten-fold diluted cDNA was used as the RT-PCR template in a 20 μL reaction system. All PCR products were loaded onto a 1% agarose gel to visualize the amplified cDNAs. RT-PCR was repeated three times. *Actin2* was used as a control for 24 cycles. The fluorescence intensity of the DNA bands was quantified using Bio-Rad's ChemiDoc^TM^ MP Imaging System. For real-time PCR, the cDNA samples were diluted to 2 ng μl^−1^. Triplicate quantitative assays were performed using 1 μL of cDNA dilution with the SYBR GreenMaster mix and an ABI fast sequence detection system according to the manufacturer's protocol (Applied Biosystems, Foster City, CA, USA). The amplification of *Actin2* was used as an internal control to normalize all data. The primers for gene expression are listed in Supplementary S2 Table at Plos one online.

### Nuclear Envelope Observation

Ten-day-old *Arabidopsis* transgenic and wild-type seedlings were treated for 0 h and 3 h at -4°C. *Arabidopsis* root tips (4–8 mm) were fixed for 5–6 h in fixation buffer (3% glutaraldehyde in 0.1 m PBS, pH 7.2). The materials were washed three or four times with 0.1 m PBS and fixed in 1% osmic acid at 4°C overnight. The materials were then washed three or four times with 0.1 m PBS and dehydrated with an ethanol series of 30, 50, 70% (4°C, overnight), followed by 80, 90, 95, and 100% (30 min for every concentration). Ethanol was then replaced with acetone (1:1) and infiltrated with a mixture of acetone: resin = 2:1 followed by mixture having a ratio of 1:1; 1:2 for 3 hours each and 100% resin for 12 h. Root tips were cut into 50–70 nm pieces using an ultramicrotome, and the nuclear envelope was observed by transmission electron microscopy (HITACHI H-7650, Japan).

### Data analysis

GraphPad primer 5 software and MS Office tools were used to create graphs and analyze the data as required. Statistical analysis was performed using Student’s t-test. P values<0.05 were considered significant.

## Results

### Molecular characterization of *AtRAN1* gene

**Fig 1:**
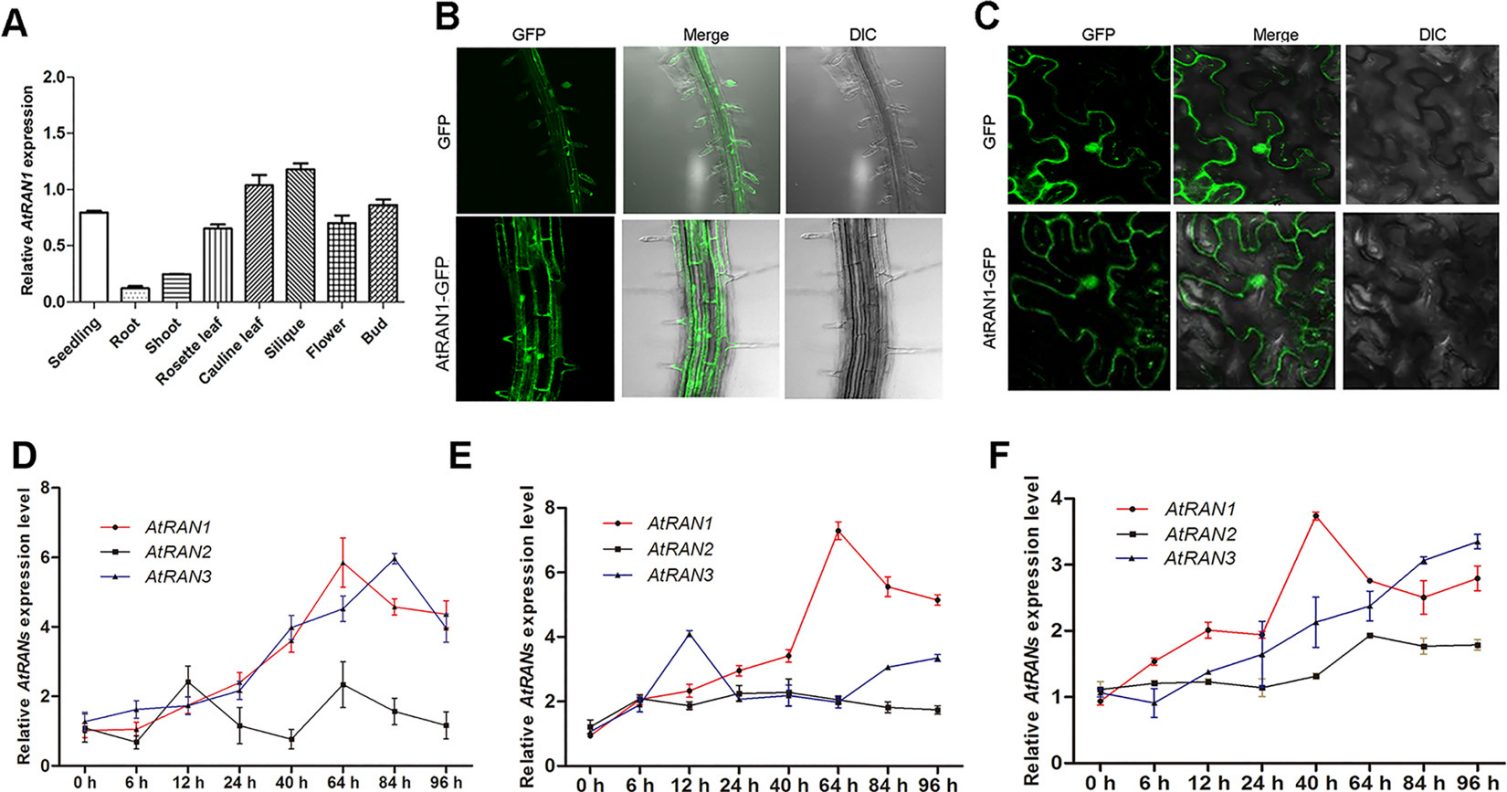
Expression Patterns and Subcellular Localization of *AtRAN1*. The results of qRT-PCR reveal. (A) Real-time PCR analysis the *AtRAN1* gene expression pattern. (B) Subcellular localization of the vector control and *AtRAN1* in transgenic *Arabidopsis* root cells. (C) Subcellular localization of the vector control, *AtRAN1* in tobacco epidermal cells. DIC, differential interference contrast, referring to bright-field images of the cells. Time course analysis of *AtRAN1*, *AtRAN2*, and *AtRAN3* expression during (D) cold acclimation (4°C). (E) Salt and (F) ABA treatment conditions. *Arabidopsis* seedlings were germinated and grown for 7 d before they were subjected to treatment. *Actin2* was used as an internal control. The error bars show SD, and are from three independent replications. And the results were repeated three times. Asterisk (*) indicates significant difference (P < 0.05).

We characterized the expression levels of the *AtRAN1* gene. The expression patterns of the *AtRAN1* gene in seedlings, roots, shoots, rosette leaves, cauline leaves, siliques, and flowers were investigated by qPCR. We found that the gene was ubiquitously expressed in plants, and the *AtRAN1* expression levels were higher in the buds, flowers, leaves, and siliques, than in the other organs examined (Fig 1A). Next, the subcellular localization of *AtRAN1*::GFP was traced to the root cells of transgenic *Arabidopsis* steadily overexpressing the *AtRAN1*::GFP gene. The green fluorescent signal of *AtRAN1*::GFP was detected mainly in the nucleus (Fig 1B), while in the control, the green fluorescent signal was randomly distributed in the cells. In addition, this localization pattern was observed through the transient expression of *AtRAN1*::GFP in tobacco epidermal cells, with identical results (Fig 1C).

We investigated the effects of abiotic stress on *AtRAN* gene expression by using qRT-PCR to monitor the expression patterns. As shown in Fig 1D, *AtRAN1* expression gradually increased after 12 h of 4°C acclimation, with the highest expression at 64 h (approximately a 6-fold increase). *AtRAN3* expression began increasing after 6 h of 4°C acclimation and reached approximately 6-fold at 84 h. The expression of *AtRAN1* increased to more than 4-fold higher after NaCl treatment, which also increased the expression of *AtRAN3* after 24 h and increased it further thereafter (Fig 1E). In addition, 0.5 μM abscisic acid was used for 96 h, with *AtRAN1* levels beginning to increase after 24 h of treatment and peaking at 64 h, with a 7-fold increase. The *AtRAN3* levels increased 4-fold 12 h after treatment and decreasing slightly thereafter (Fig 1F). In conclusion, the data suggest that *AtRAN1* and *AtRAN3* respond to cold, salt and ABA treatment in different degrees.

To address the function of the *AtRAN* genes, T-DNA insertion mutants were obtained from the SALK T-DNA insertion collection. Seeds of *AtRAN1-1* (SALK_138680), *AtRAN1-2* (SALK_067649), *AtRAN2* (SALK_123620c), *AtRAN3* (SALK_074683) plants were obtained, and homozygous mutants were verified using diagnostic PCR screening and DNA sequencing. *AtRAN* gene expression in the mutants was checked by qRT-PCR. Our data verified the insertion and a large reduction in *AtRAN1* expression in the SALK_138680 line and the SALK_067649 line. Furthermore, *AtRAN2* gene expression decreased in the SALK_123620c mutant. The SALK_074683 mutant showed no *AtRAN3* expression (S1 Fig). Because the *AtRAN1* and *AtRAN2* genes are linked on the same chromosome, the *atran1* and *atran2* mutants were subsequently crossed with *atran3* to obtain homozygous *atran1 atran3*, *atran2 atran3* double mutants. We overexpressed *AtRAN1* in the wild-type under the control of the CaMV 35S promoter (S1 Fig).

### *AtRAN1* overexpression promotes vegetative growth and increases yields

**Fig 2:**
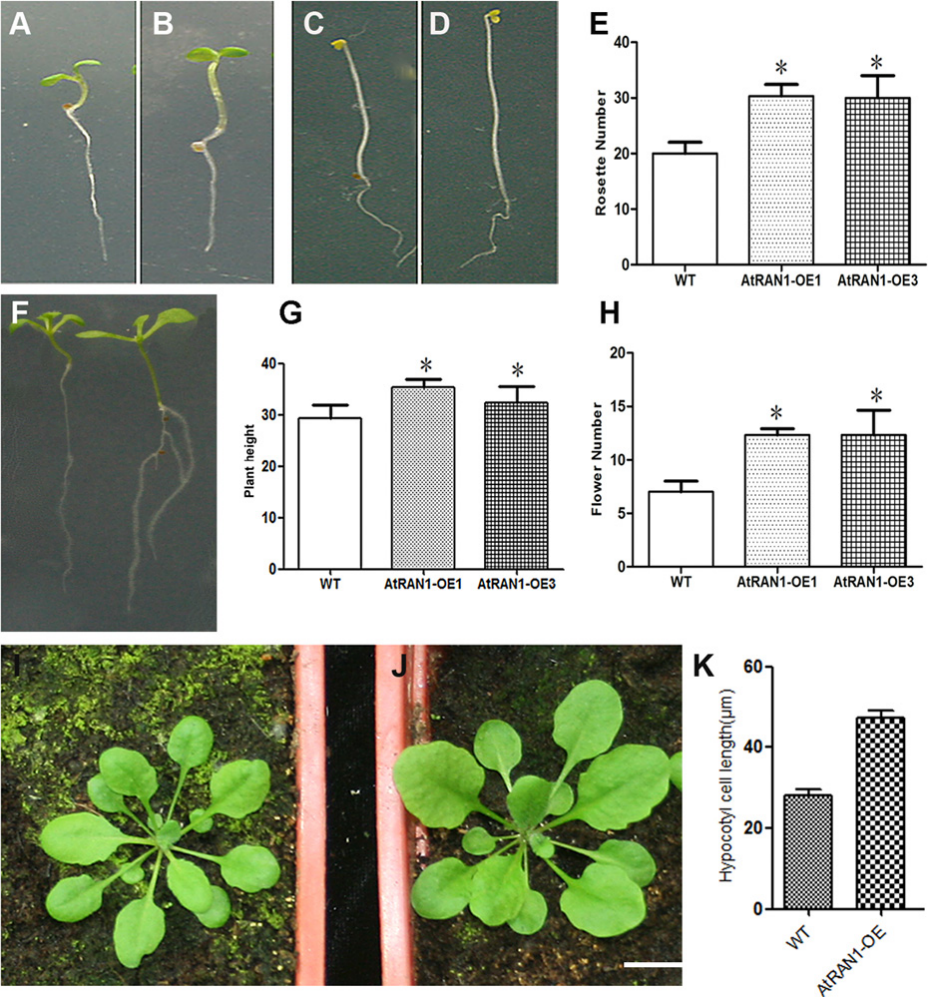
Pleiotropic Phenotype of *AtRAN1* overexpressing Plants. Seven-day seedlings hypocotyls in Wild-type (A, C) and *AtRAN1*-OE1 plants (B, D) transgenic lines plants under white light and dark conditions; Analysis of (E) Rosette leaf number; (F) Root phenotype of WT and transgenic plants. (G) Plant height; (H) Flower number; Five-week seedlings of Wild-type (I) and *AtRAN1*-OE1 plants (J). (K) Hypocotyl cell length in dark grown seedlings. Flower number; Figures I, J Bars = 0.5 cm. The error bars show SD.

To characterize the *AtRAN1* gene function in *Arabidopsis*, we first obtained many lines of highly overexpressing transgenic plants. The *AtRAN1-*OE plants showed many phenotypic differences during plant development. Under long-day conditions, the transgenic seeds exhibited a quicker germination rate, the transgenic 7-day seedling hypocotyls were much longer than in wild-type. Moreover, *AtRAN1-*OE plants exhibited larger leaf size and longer siliques (Fig 2, Table 1). In root development, the *AtRAN1-*overexpressing plants had approximately 1.5 cm longer primary roots and approximately 5 more lateral roots than the 16-day-old wild-type seedlings (Table 1). Thus, *AtRAN1* overexpression promotes plant vegetative growth. Furthermore, we isolated the *atran1-1*, *atran1-2*, and *atran1-1 atran3* mutants. However, no obvious differences were manifested in the leaf development in comparison with the wild-type plants. This rather weak mutant developmental phenotype may be due to the unattainability of the *AtRAN1* knockout mutant or/and the functional redundancy with other Ran genes.

**Table 1:** Seed production and root phenotypes in different genotype plants.

Genotype	Seed weight (mg/plant)	Silique length (cm)	Main root (cm)	Lateral root number
**Col-0**	366 ± 55	1.6 ± 0.2	7.3 ± 0.8	13.3 ± 1.2
***atran1-1***	342 ± 29	1.44 ± 0.4	6.8 ± 0.7	10.3 ± 1.7^a^
***atran1-2***	338 ± 42	1.47 ± 0.6	7.1 ± 1.3	11.0 ± 0.8^a^
***atran1-1 atran3***	321 ± 38^a^	1.35 ± 0.1	6.4 ± 0.6	9.6 ± 0.7^a^
***atran1-2 atran3***	324 ± 31^a^	1.38 ± 0.2	6.6 ± 0.4	9.1 ± 0.9^a^
***AtRAN1*-OE1**	431 ± 46^a^	1.89 ± 0.3^a^	8.9 ± 1.1^a^	16.8 ± 1.3^a^
***AtRAN1*-OE3**	433 ± 41^a^	1.87 ± 0.3^a^	8.8 ± 0.7^a^	17.9 ± 0.9^a^

Plants were grown to full maturity in greenhouse conditions. The weight of seeds harvested was measured for each plant. The main root length and lateral root number were measured in 16-day-old seedlings on the MS medium. The mean values ± SD of seven independent plants are shown.

^a^ indicates value that are significantly different from the respective controls (P<0.05). ND; not determined.

In addition to the promotion of vegetative growth by *AtRAN1*, we also characterized the seed yields in the mutants and overexpression lines. We observed that seed production and germination rate (S4 Fig) were much higher in the plants overexpressing *AtRAN1*. This difference may be due to increased plant growth and fitness and the resulting increases in both seed and silique size (Table 1). It has previously been reported that decreased levels of *AtRAN1* affect seed body size in *Arabidopsis* [12]. Thus, the *AtRAN1* gene both promoted plant growth and increased seed production in *A*rabidopsis.

### *AtRAN1* regulates salt tolerance and ABA insensitivity redundantly with *AtRAN3*

**Fig 3:**
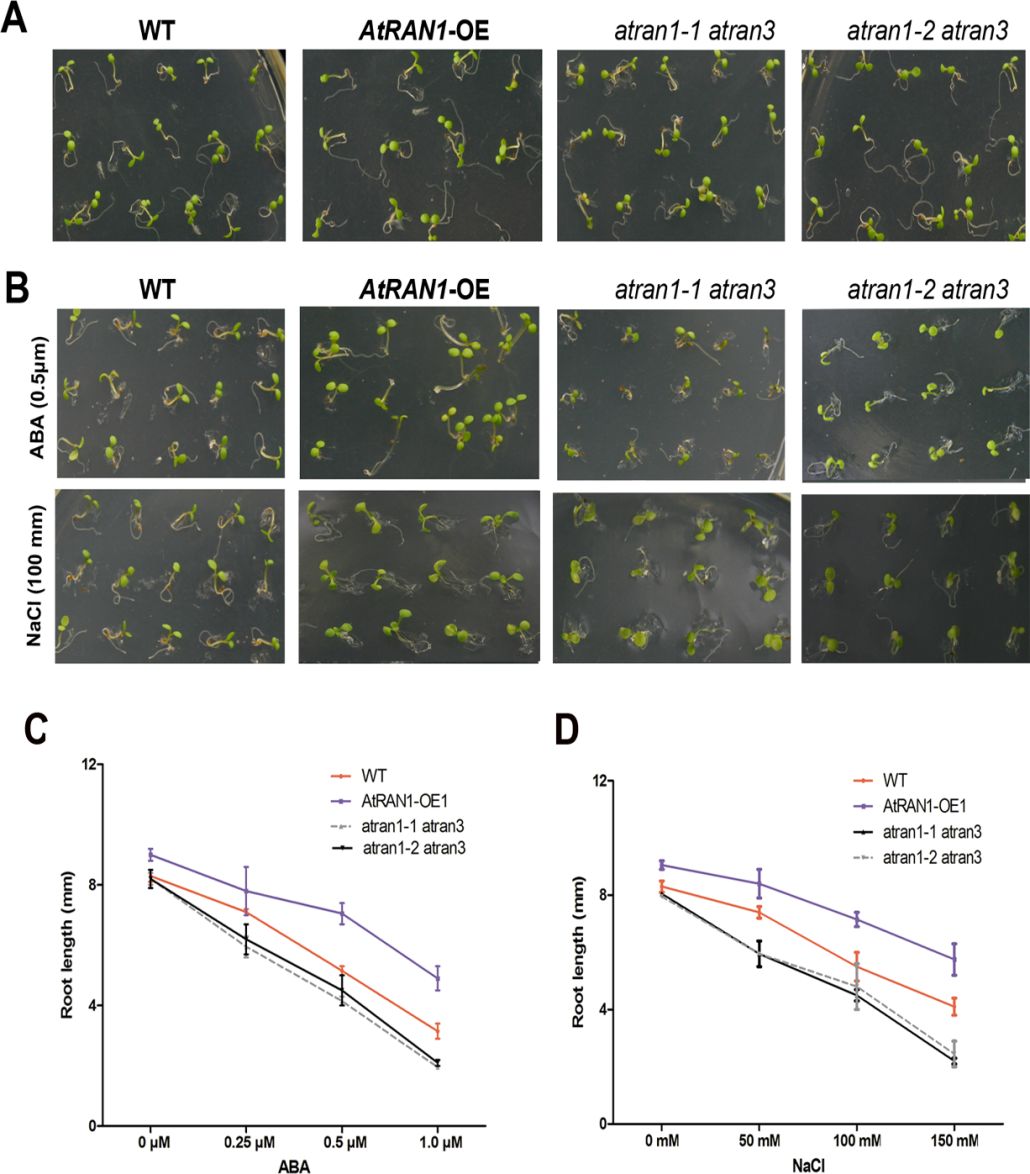
Analysis of salt stress and ABA tolerance of RAN1 overexpression plant and *atran1 atran 3* double mutant. (A) *atran1-1 atran3*, *atran1-2 atran3* mutants, *AtRAN1*-OE1, and the wild-type grow on MS medium supplemented with 1% sucrose. Photos were taken 7 days after germination. (B) Plants were grown on MS medium supplemented with 1% sucrose and 0.5 μM ABA (upper panel) or 100 mM NaCl (lower panel), respectively. Photos were taken 5 days after treatment. (C) and (D) Dose response curve of root growth under ABA (C), or salt stress conditions (D). Plants were germinated on MS medium supplemented with 1% sucrose. Root lengths were measured 5 days after ABA or NaCl treatment.

Because the *AtRAN1* and *AtRAN3* genes are responsive to abiotic stress, we wanted to determine whether the *atran1 atran3* double mutant plants show the growth phenotype under the condition of salt stress and exogenous ABA treatment. Under normal conditions, *atran1 atran3* seed germination was identical to wild-type (Fig 3A). Under salt and ABA treatment, compared with the *atran1*, *atran2*, *atran3* and wild-type plants, both the *atran1-1 atran3* and *atran1-2 atran3* double mutant seedlings showed higher sensitivity than the wild-type (Fig 3B and S3 Fig). Since the overexpression plants are already faster to grow and bigger than wild-type and mutants, we also do a dose response curve for post germination growth in the presence of ABA and salt treatment (Fig 3C and 3D). The results showed that the inhibition effects of ABA were smaller on *AtRAN1*-OE plants than on *atran1atran3* double mutant and wild-type. So we get the conclusion that *AtRAN1* overexpression increased the tolerance to ABA treatment. Thus, we concluded that *AtRAN1* was involved in salt tolerance and the ABA signaling pathway. The results suggested that *AtRAN1* was required for the ABA-mediated response to general osmotic stress during early seedling growth.

### *AtRAN1* overexpression altered freezing tolerance in *Arabidopsis*

**Fig 4:**
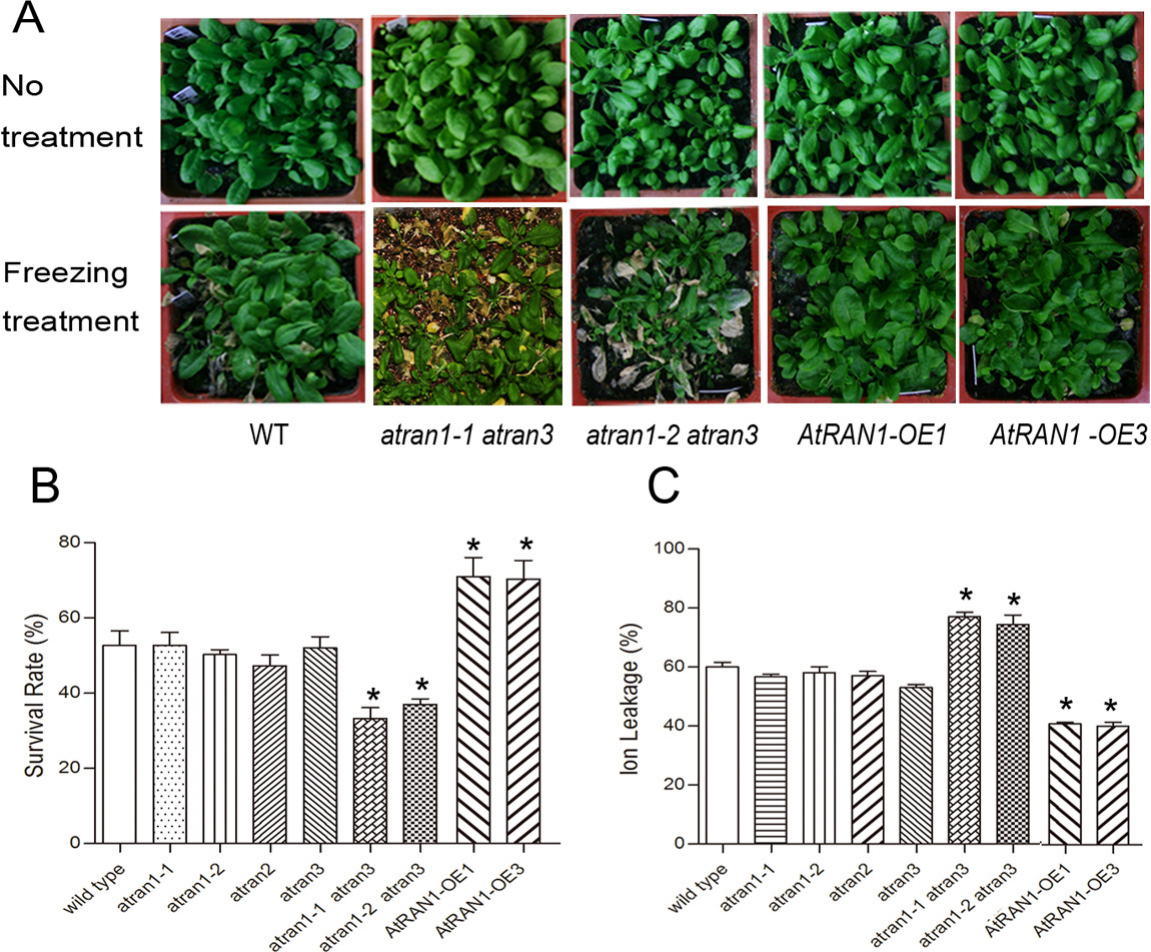
Freezing Tolerance Analysis of *atran* Mutants and transgenic plants. (A) Three-week-old *AtRAN* gene double mutants and WT plants were cold stressed at -14°C after 4-day 4°C acclimation and then transferred back to the normal condition for recovery. Photographs of representative seedlings of the WT and transgenic lines were taken after 14 d of recovery. (B) Survival rate of the mutants and transgenic plants after freezing stress. (C) Ion leakage assay of the mutants and transgenic plants after freezing stress. The error bars show SD, and were from three independent replications. And the results were repeated three times. Asterisk (*) indicates significant difference (P < 0.05).

Enhanced sensitivity to freezing was also observed in the *atran1 atran3* double mutant. The freezing tolerance of the *atran1*, and *atran3* single mutants, the *atran1-1 atran3* and *atran1-2 atran3* double mutants and *AtRAN1-*OE were compared with wild-type plants after 4°C acclimation in growth chambers. Fig 4A shows the status of each leaf in a pool of plants of each genotype. Representative plants of each population were used to visualize the phenotype before and after freezing treatment. The figure clearly shows that the *atran1-1 atran3* and *atran1-2 atran3* double mutants exhibited greater freezing susceptibility, with 36% and 39% survival rates. In contrast, the survival rates of the *atran1*, *atran3* single mutants were not significantly different from the wild-type after the -14°C freezing treatment. The ion leakage was higher in the *atran1 atran3* mutant background (Fig 4C). Meanwhile, *AtRAN1-*OE plants exhibited increased freezing tolerance under cold acclimated conditions compared to the wild-type plants (Fig 4B). Specifically, the survival rate of the wild-type plants treated after 4°C acclimation was 52%, but *AtRAN1-*OE lines showed a survival rate of 74% after freezing treatment. In addition, the ion leakage was lower in the *AtRAN1*-OE lines. Freezing tolerance was also determined for a range of different freezing temperatures for each transgenic line, and the LT50 (i.e., the temperature at which 50% of plants survive) was determined (S2 Fig). These data supported the hypothesis that *AtRan1* positively regulates freezing tolerance.

### *AtRAN1* regulates cold response gene expression levels

**Fig 5:**
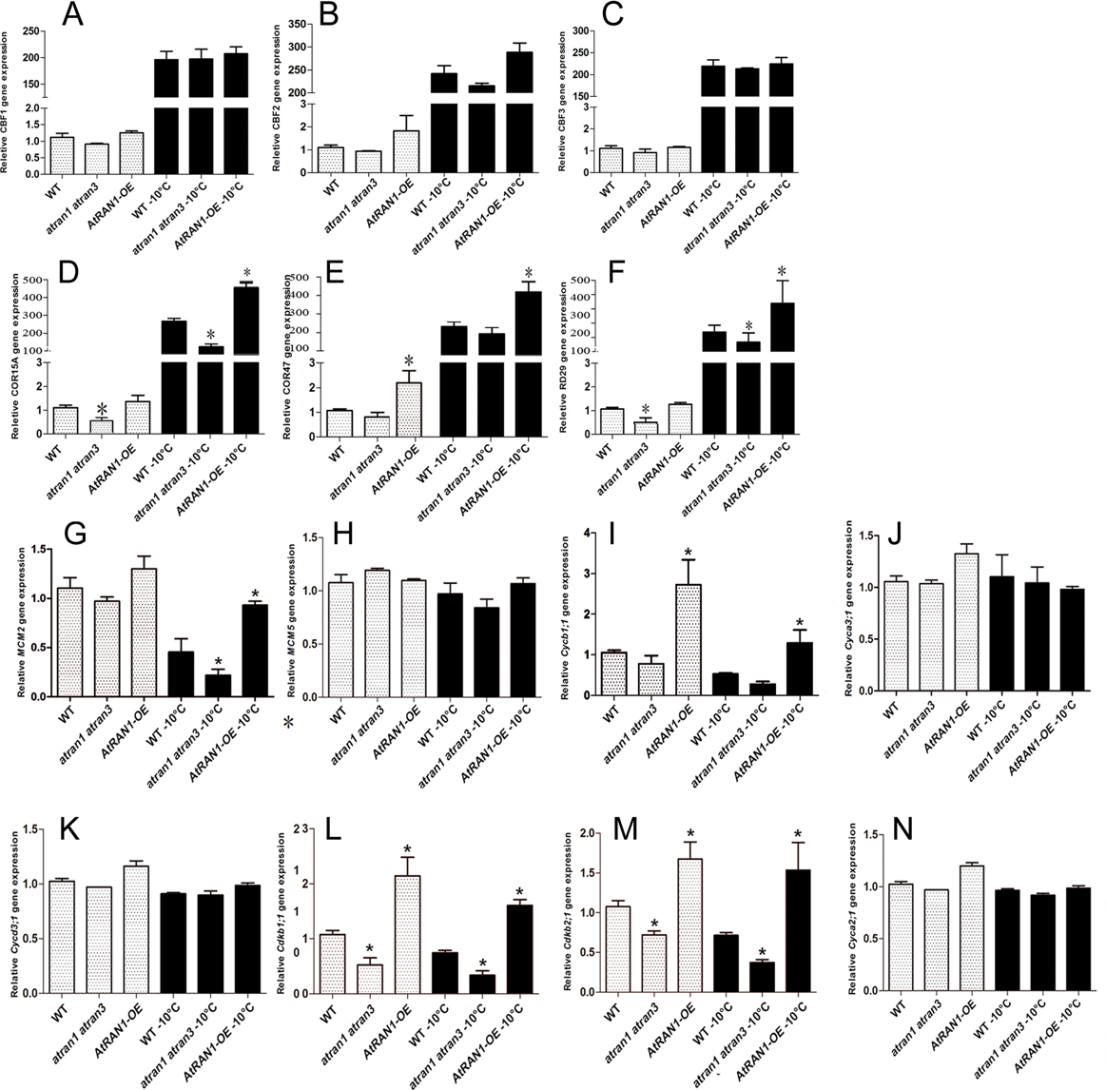
Cold response Genes and Cell Cycle-related Genes expression Under Normal and Freezing Conditions. Expression levels of (A) *AtCBF1* (DREB1B), (B) *AtCBF2* (DREB1C) and (C) *AtCBF3* (DREB1A) genes and (D) COR15A, (E) COR47 and (F) RD29A downstream genes in 7-day old *AtRAN1*-OE, the *atran1-1 atran3* mutant and wild-type plants under before and after freezing treatment. Values are means and SD (n = 4). Expression of (G) *MCM2*, (H) *MCM5*, (I) *Cycb1;1*, (J) *Cyca3;1*, (K) *Cycd3;1*, (L) *Cdkb1;1*, (M) *Cdkb2;1* and (N) *Cyca2;1* cell cycle-related gene levels in wild-type, *atran1-1 atran3* mutant and transgenic plants before and after 0.5h freezing stress, after 4°C acclimation, The error bars show SD, and are from three independent replications. And the results were repeated three times. Asterisk (*) indicates significant difference (P < 0.05).

Cold stress induced the *CBF* genes, which are well-known regulators of cold response. CBF proteins activated the transcription of the DRE/CRT *cis*-element-containing COR genes [42–44]. To determine the molecular mechanisms by which *AtRAN1* regulated freezing tolerance, we tested changes in the expression of the cold responsive genes. When *atran1-1 atran3*, *AtRAN1-*OE1 and wild-type plants were treated after freezing, the expression levels of *CBF1* and *CBF3* were increased to identical levels, but *CBF2* expression levels were lower in the *atran1 atran3* mutant and slightly higher in *AtRAN1-*OE1 (Fig 5A–5C). The expression levels of downstream genes, including *COR15A*, *COR47*, and *RD29A*, were much lower in the *atran1 atran3* mutant after freezing treatment but higher in the *AtRAN1-*OE1 plants (Fig 5D–5F). These results suggest that *AtRAN1* modulates cold signaling pathway components.

### *AtRAN1* overexpression promotes cell cycle genes expression

To further illuminate the cell cycle-related genes involved in the control of cold signaling, we also examined the expression patterns of genes related to the cell cycle. Compared with the expression levels of the type A and D cyclin genes, the expression levels of the type B cyclin gene *Cycb1;1*, the type B cyclin gene related kinase (*Cdkb1;1*, *Cdkb2;1*) and *MCM2* were suppressed by short-term freezing treatment in both the wild-type and mutant plants. In the *AtRAN1-*OE line, the expression levels of *Cycb1;1*,*Cdkb1;1*,*Cdkb2;1*,*MCM2* were much higher than in the wild type and mutant plants both before and after the freezing treatment (Fig 5I, 5L and 5M). Thus, cold responsive genes and cell proliferation-related genes are involved in the *AtRAN1*-mediated cold tolerance pathway.

### *AtRAN1* regulates nuclear envelope maintenance under cold

**Fig 6:**
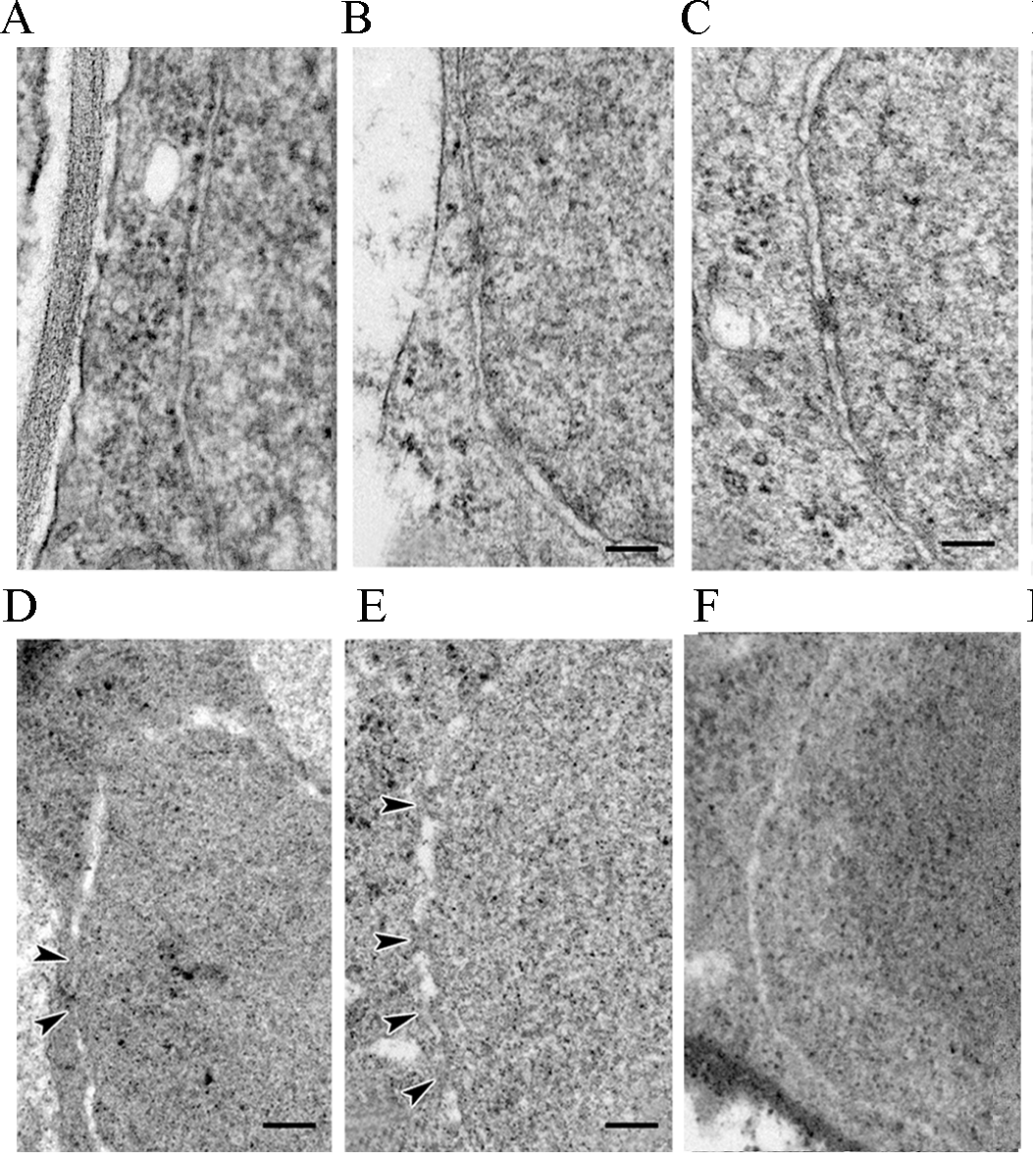
Morphological Changes in Nuclear Envelope under Normal and Freezing Conditions. Nuclear envelope of the (A) WT, (B) *atran1-1 atran3* and (C) *AtRAN1*-overexpressing plants under normal conditions (22°C). After 4-day 4°C acclimation, Nuclear envelope of the (D) WT, (E) *atran1-1 atran3* and (F) *AtRAN1*-overexpressing lines were treated for 2h at -4°C. Six root tips were observed in every condition. The root tips were transversely cut in the meristematic zones. Arrows indicate the abnormal nuclear envelope. Bars = 100 nm. The error bars show SD, and are from three independent replications. The results were repeated three times.

We observed the nuclear envelopes of root tip cells in the *AtRAN1-*OE, *atran1 atran3* and wild-type *Arabidopsis* lines under normal and freezing conditions. Under the normal condition (22°C), the nuclear envelope NE was intact in all four lines, with no obvious differences in morphology (Fig 6A–6C). After freezing treatment at -4°C for 2 h, approximately 60%-70% of wild-type cells showed partially dissociated double membranes (Fig 6D). Most of the cells in the transgenic lines showed an intact nuclear envelope (Fig 6F). However, the atran1-1 a*tran3* double mutant showed many fragmentized nuclear envelopes, with few cells showing slightly dissociated nuclear envelopes (Fig 6E). These results suggested that *AtRAN1* overexpression promotes the formation of an intact nuclear envelope under freezing conditions.

## Discussion

### *AtRAN1* function is well-conserved in cell cycle regulation

Ran proteins coupled with importins and exportins transport cargo proteins between the nucleus and cytoplasm. Also RanGTP plays an important role during mitosis [45]. Ran participates in various cellular processes by means of the gradient mechanism, and it concentrates around action site [46]. The nuclear envelope (NE) is a relatively isolated layer and provides a special space for gene transcription and protein translation. The nuclear envelope undergoes dynamic changes during the cell cycle progression [47–48]. Early in the mitosis stage, Ran separates from chromatin to the cytoplasm. During anaphase to telophase, coupled with nuclear envelope assembly, Ran GTPase promotes nuclear pore organization in *Xenopus* egg extracts [40]. In plants, despite previous reports on the function of *OsRAN1* and *OsRAN2* in nuclear envelope assembly, our observations indicated that *AtRAN1* overexpression also promoted the formation of an intact nuclear envelope and cell cycle progression under freezing conditions (Fig 6). Therefore, plant Ran homologs may have functions that are conserved in plants, yeast, and animals during evolution.

Although *AtRAN1* and *AtRAN3* are both induced by cold, salt, and ABA treatment, *AtRAN1* shows a high homology to *AtRAN3*. However, *AtRAN1-*OE plants exhibit many developmental phenotypes different from *AtRAN3-*OE plants. Consistently with this hypothesis, previous results have revealed that the expression level of the *AtRAN1* gene is approximately 2-fold higher than *AtRAN3*. The physiological functions of the *Ran* genes in monocot and dicot plants are not always identical. Others have reported that *OsRAN2* overexpression in rice and *Arabidopsis* resulted in enhanced sensitivity to osmotic stress and ABA [18]. *TaRAN* and *OsRAN1* overexpression in *Arabidopsis* was found to inhibit root development [20]. Therefore, apart from the conserved role of the Ran function in cold resistance, *Ran* genes do not always function identically in plant development.

### *AtRAN1* links plant growth and stress tolerance with cell proliferation

To gain information about the biological role of plant RanGTPase proteins in *Arabidopsis*, we obtained Ran mutants and overexpression lines to reveal gene functions. The *AtRAN1 -*overexpressing transgenic plants manifested increased plant growth, whereas the *atran1 atran3* double mutant caused some developmental defects. The hypocotyl elongation of seedlings was affected by *AtRAN1* overexpression under white light and dark conditions, suggesting that the *AtRAN1* control of hypocotyl cell elongation is independent of light. *AtRAN1* overexpression plants hypocotyl cell were longer that the control (Fig 2K). The leaf sizes were larger in the *AtRAN1* overexpression plants. We speculate that *AtRAN1* is involved in plant growth through promoting both cell division and cell expansion.

Many studies have shown that cell division is closely related to stress tolerance. Overexpressing *HAL3a* showed improved growth as well as salt and osmotic tolerances in *Arabidopsis* and rice [49]. Transgenic rice lines overexpressing *OsMYB3R-2* and *OsCycB1;1* exhibited enhanced cold tolerance by maintaining cell division activity under cold condition [50]. Our results indicate that the expression of *AtRAN1* was up-regulated under cold stress (Fig 1D). This expression pattern suggests that *AtRAN1* may function as a regulator in the cold signaling pathway in *Arabidopsis*. In addition, *AtRAN1-*OE plants showed increased freezing tolerance after 4°C acclimation. Furthermore, we observed differences in freezing tolerance between the *atran1 atran3* mutant and wild-type plants (Fig 5). These data demonstrated that *AtRAN1* positively regulated freezing tolerance by maintaining the cell cycle. By combining our results with the findings of previous studies, we speculate that *AtRAN1* is an important regulatory link between plant growth and environmental cues.

## Supporting Information

S1 Fig Isolation of *AtRAN* mutants and obtaining of transgenic lines. (A) Structure of genomic clones encoding the *AtRAN1*, *AtRAN2*, and *AtRAN3* proteins.(B) *Arabidopsis* T-DNA mutant screen. (C) The expression pattern of *AtRAN* genes in the mutant background. (D) Real-time RT-PCR analysis of the expression of *AtRAN1* (E) Real-time RT-PCR analysis of the expression of *AtRAN3*.
(TIF)

S2 Fig Survival rates of cold-acclimated plants after freezing treatment. Percent survival of acclimated three-week-old *AtRAN1*-OE lines, *atran1 atran3* double mutant and Wild-type plants were frozen in a temperature-controlled chamber as described under Methods. At the temperatures shown, samples of plants were removed from the chamber, allowed to recover, and scored for survival. The data are means ± SE for three separate experiments.
(TIF)

S3 Fig Analysis of salt stress and ABA tolerance of RAN1 overexpression plant and *atran1atran3* double mutant. Treatment conditions are same as those in Fig 3, except that plants were germinated on MS medium without sucrose. The AtRAN1-OE1 transgenic plants show higher resistance to ABA and salt treatment than the wild-type, while atran1-1 atran3 and atran1-2 atran3 show increased salt sensitivity than the wild-type.
(TIF)

S4 Fig Germination rates and dose response curve for post germination growth of wild type, *AtRAN1*-OE plants and *atran1atran3* double mutant. (TIF)

S1 Table Primers used in plasmid construction. (DOCX)

S2 Table Gene-specific primers used in qPCR experiments. (DOCX)

S3 Table Primers used in mutant isolation. (DOCX)

S4 Table Reported Ran gene functions from various plants. (DOCX)
