# Supplementary figures and images for: The Small G Protein AtRAN1 Regulates Vegetative Growth and Stress Tolerance in Arabidopsis thaliana
Source: PLoS One. 2016 Jun 3;11(6):e0154787. doi: 10.1371/journal.pone.0154787 (PMC4892486; doi:10.1371/journal.pone.0154787)

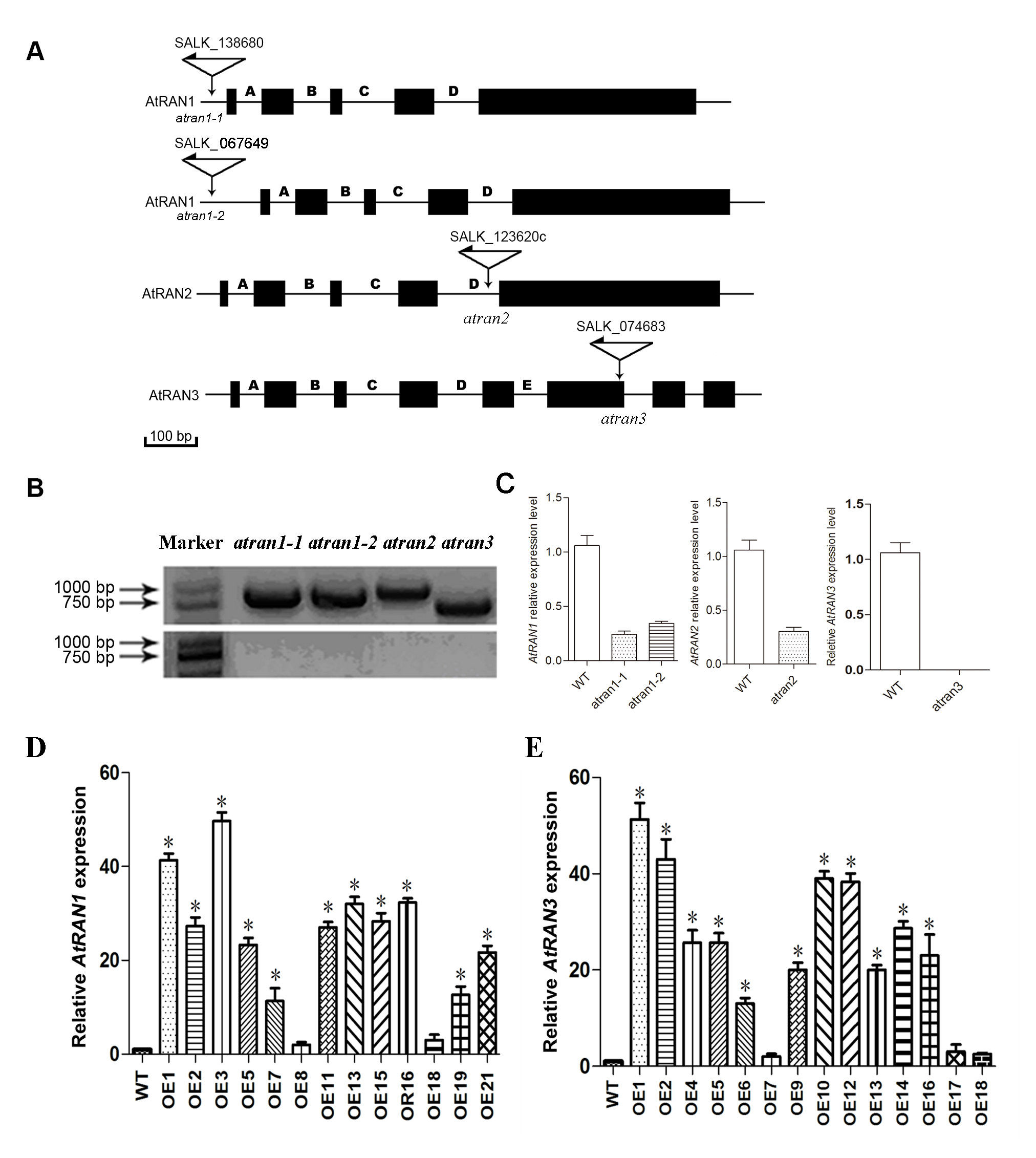

Supplement: S1 Fig — (A) Structure of genomic clones encoding the AtRAN1, AtRAN2, and AtRAN3 proteins.(B) Arabidopsis T-DNA mutant screen. (C) The expression pattern of AtRAN genes in the mutant background. (D) Real-time RT-PCR analysis of the expression of AtRAN1 (E) Real-time RT-PCR analysis of the expression of AtRAN3. (TIF) [file pone.0154787.s001.tif]

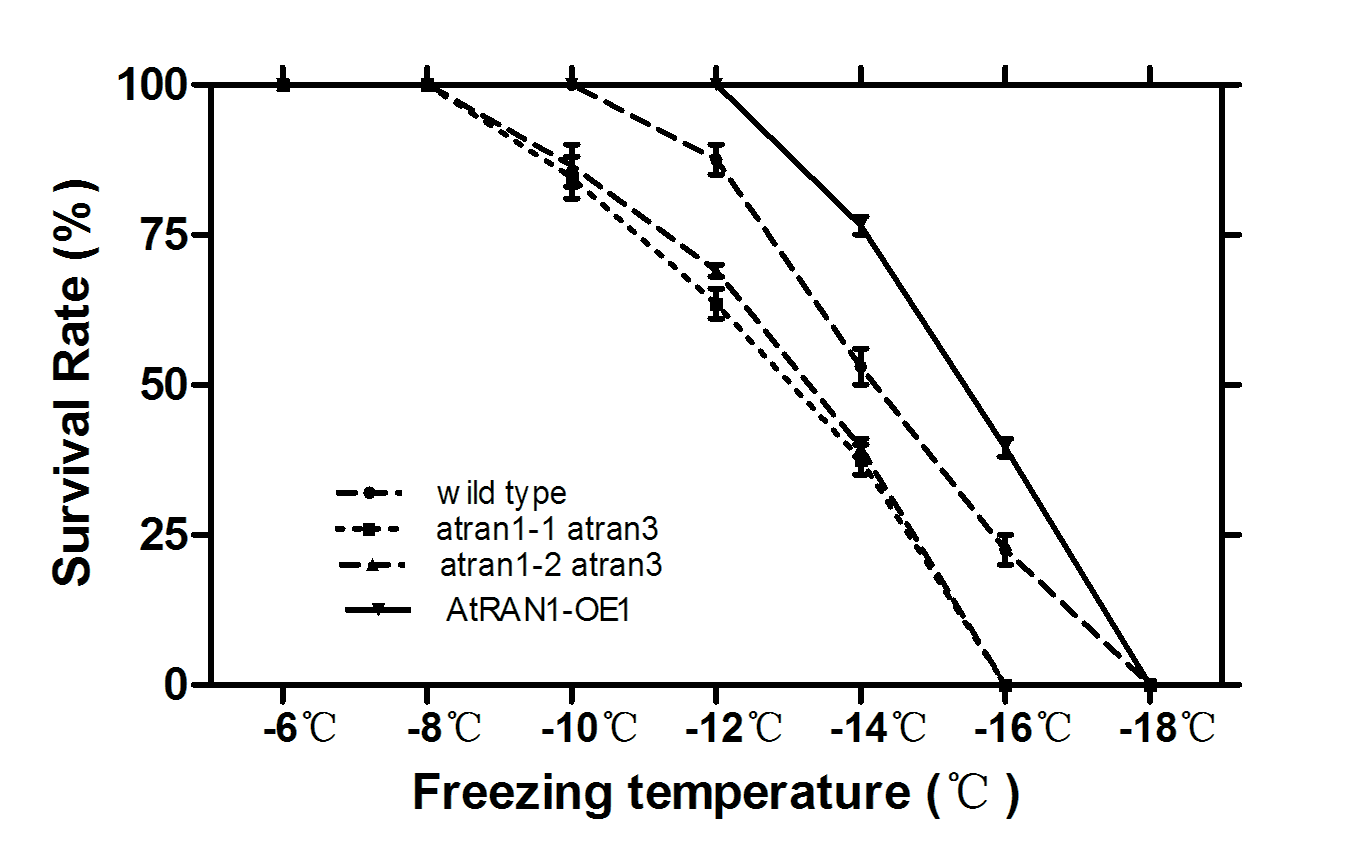

Supplement: S2 Fig — Percent survival of acclimated three-week-old AtRAN1-OE lines, atran1 atran3 double mutant and Wild-type plants were frozen in a temperature-controlled chamber as described under Methods. At the temperatures shown, samples of plants were removed from the chamber, allowed to recover, and scored for survival. The data are means ± SE for three separate experiments. (TIF) [file pone.0154787.s002.tif]

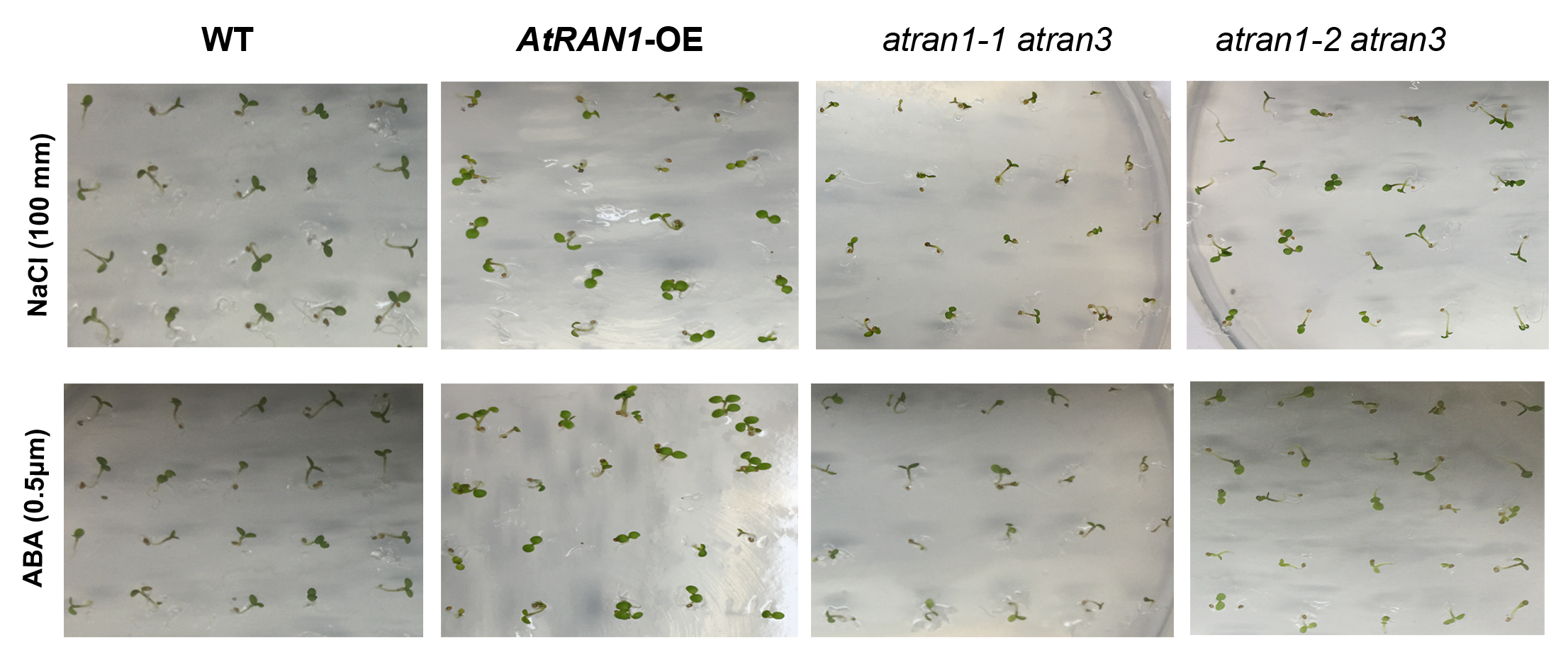

Supplement: S3 Fig — Treatment conditions are same as those in Fig 3, except that plants were germinated on MS medium without sucrose. The AtRAN1-OE1 transgenic plants show higher resistance to ABA and salt treatment than the wild-type, while atran1-1 atran3 and atran1-2 atran3 show increased salt sensitivity than the wild-type. (TIF) [file pone.0154787.s003.tif]

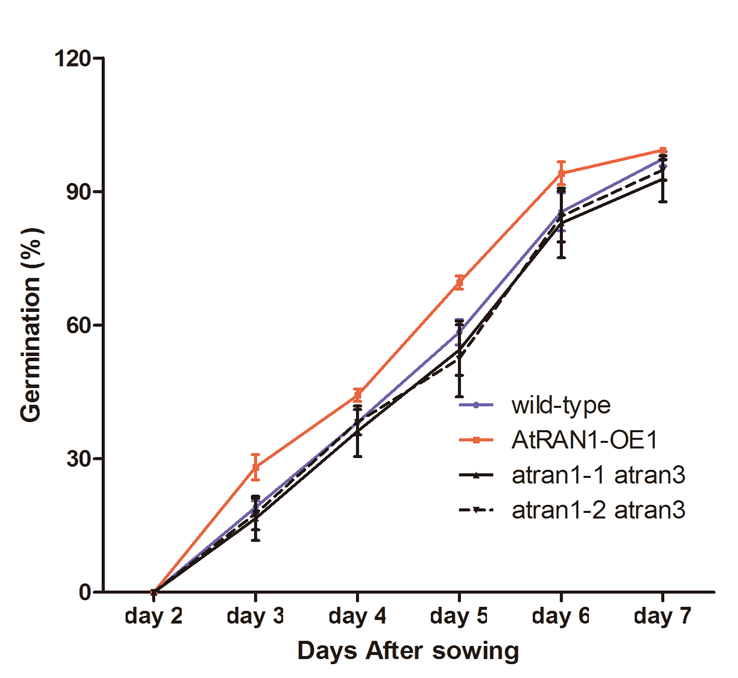

Supplement: S4 Fig — (TIF) [file pone.0154787.s004.tif]
