# Supplementary material for: The Small G Protein AtRAN1 Regulates Vegetative Growth and Stress Tolerance in Arabidopsis thaliana
Source: PLoS One. 2016 Jun 3;11(6):e0154787. doi: 10.1371/journal.pone.0154787 (PMC4892486; doi:10.1371/journal.pone.0154787)
Supplement: S1 Table — (DOCX) [file pone.0154787.s005.docx]

**S1 Tab. Primers used in plasmid constructions**

| **Transgenes** | **Primers (Sequence 5’-3’)** |
| --- | --- |
| Clone *AtRAN1* cDNA | 5'-ATCTTCATTCTCCCTCTCGCCG-3′^a^ |
|  | 5'-AACTGGCAAGTAATAGGAGGTA-3′^a^ |
| Clone *AtRAN3* cDNA | 5'-CGTCATTCCTAAGCTCACTATA-3′^a^ |
|  | 5'-TGAAATTGTGAAGCATGTAAGG-3′^a^ |
| PER8-*AtRAN1* | 5'-CTCGAG ATGGCTCTACCTAACCAGCA-3′ |
|  | 5'-ACTAGTCTCAAAGATATCATCATC-3′ |
| double 35S:*AtRAN1*-HA | 5'-GGATCC ATGGCTCTACCTAACCAGCA-3′ |
|  | 5'-CTAGA**AGCGTAATCTGGAACATCGTATGGGTA** CTCAAAGATATCATCATC-3′ |
| double 35S:*AtRAN3*-HA | 5'-AAGCTT ATGGCTCTACCTAACCAGCA-3′ |
|  | 5'-TCTAGA**AGCGTAATCTGGAACATCGTATGGGTA** CTCGAAGGTGTCATCA-3′ |
| double35S:*AtRAN1*:GFP | 5'-GGAATTCATGGCTCTACCTAACCAGCA-3′ |
|  | 5'- GGTCGACCTCAAAGATATCATCATC-3′ |
|  | 5’-GGTACCATGGTAGATCTGACTA-3’ ^b^ |
|  | 5’GAGCTCTCACACGTGGTGGTGGT-3’ ^b^ |

Underlined are restriction digestion sites.

Sequence shown in bold is used to clone HA-tag.

a: primers designed according to 5’/3’-UTR used for clone Ran gene cDNAs in *Arabidopsis*.

b: specific primers for GFP.
