# Supplementary material for: The Small G Protein AtRAN1 Regulates Vegetative Growth and Stress Tolerance in Arabidopsis thaliana
Source: PLoS One. 2016 Jun 3;11(6):e0154787. doi: 10.1371/journal.pone.0154787 (PMC4892486; doi:10.1371/journal.pone.0154787)
Supplement: S2 Table — (DOCX) [file pone.0154787.s006.docx]

**S2 Tab. Gene-specific primers used in qPCR experiments**

| **Genes** | **Primers (Sequence 5’-3’)** |
| --- | --- |
| ACTIN2 | 5’-TTCCCGTTCTGCGGTAGTGG-3’ |
|  | 5’-CCGGTATTGTGCTCGATTCTG-3’ |
| AtRAN1 | 5'-ATCGCCTGAATTCGCTCTCC-3′^a^ |
|  | 5'-TAGTCTTCCCTGTGCCTCCA-3′^a^ |
|  | 5'-GGAGGCACAGGGAAGACTAC-3′^b^ |
|  | 5'-TCTCTTGTCCAGCAGTGTCC-3′^b^ |
| AtRAN2 | 5'-GCCGCATCGCTTCTTAAACC-3′^a^ |
|  | 5'-GCCTCCATCACCAACGATGA-3′^a^ |
| AtRAN3 | 5'-TCCCCCGGAAGTTCAAATCG-3′ |
|  | 5'-TTCAGACAAGCCCTAACGGG-3′ |
| AtRAN4 | 5'-TGCTGGAGACGCAAAACTTTC-3′ |
|  | 5'-TAGGAGTGGCTGAGTTCCCG-3′ |
| CBF1 | 5'-ACCAAAAGAAGAGTCCTAATCCG-3' |
|  | 5'-TTCGGACAACTCGTGGCCAA-3' |
| CBF2 | 5'-ATATGGATGAAGAGGCGATGTTGG-3' |
|  | 5'-TTCGGACAACTCGTGGCCAA-3' |
| CBF3 | 5'-CTGAAGCTGCGTTGGCGTTT-3' |
|  | 5'-TCTCCTCCATGTCGAAGCCA-3' |
| COR15A | 5'-CAGTGAAACCGCAGATACATTGGG-3' |
|  | 5'-CGGCTTCTTTTCCTTTCTCCTCC-3' |
| RD29A | 5'-TGATCGATGCACCAGGCGTAA-3' |
|  | 5'-CCCTGGTGGAATAATTTCCTCCG-3' |
|  | 5’-ACAGCTGGTGAATCCTCTGC-3’ |
| MCM2 | 5'-ACGAGAATCAATGAACGGAC-3’ |
|  | 5'-CAGACATTCGGATCATGGAC-3’ |
| MCM5 | 5'-AGGCTAATGAGGGAGGGGTA-3’ |
|  | 5'-GGAACTGGCCTCATTTGTGT-3’ |
| CYCA2;1 | 5'-ACGTTTCTCAGGCGGTTCAT-3’ |
|  | 5'-TGGTCGAGTGTCCATCTTGC-3’ |
| CYCA 3;1 | 5'-CATTCTTTCAAATCACCCGC-3’ |
|  | 5'-CTAGGTTCATCATCCGTCCA-3’ |
| CYCB 1;1 | 5'-GCTTCTGCAATCTACGCAGC-3’ |
|  | 5'-CCAACAGCTTTGCACAGTCC-3’ |
| CYCB 2;1 | 5'-CGAGAAGATGAGAGCAATAC-3’ |
|  | 5'-TCGATGAGCCAGTCAATAAG-3’ |
| CYCD 3;1 | 5'-GCAAGTTGATCCCTTTGACC-3’ |
|  | 5'-CAGCTTGGACTGTTCAACGA-3’ |
| CDKB 1;1 | 5'-TGAGATGGTTCGGAGGCAAG-3’ |
|  | 5'-AGTCACGCAGTGTGGAAACA-3’ |
| CDKB 2;1 | 5'-CACGTCGTCAGGTTAATGGA-3’ |
|  | 5'-TGTTCTTGCCAGTGCTACGG-3’ |

^a^ primers used for Real-time PCR in Figures 1.

^b^ primers used for Real-time PCR in Figure S1,S2,S3.
