# Supplementary material for: The Small G Protein AtRAN1 Regulates Vegetative Growth and Stress Tolerance in Arabidopsis thaliana
Source: PLoS One. 2016 Jun 3;11(6):e0154787. doi: 10.1371/journal.pone.0154787 (PMC4892486; doi:10.1371/journal.pone.0154787)
Supplement: S3 Table — (DOCX) [file pone.0154787.s007.docx]

**S3 Tab.** **Primers used in mutant isolation**

| **Genes** | **Mutants** | **Primers** |
| --- | --- | --- |
| AtRAN1 | SALK_138680 | 5′-TTGGTTATTACGGTCAGGCTG-3′ |
|  |  | 5′-CGAAGAGTACCGTACCTGTGC-3′ |
| AtRAN1 | SALK_067649 | 5′-ATTCTTGTATGTGAGCCGTGC-3′ |
|  |  | 5′-CCTCTCCATCTTCATTCTCCC-3′ |
| AtRAN2 | SALK_123620C | 5′-CCTAAGGCCACCAAATTTCTC-3′ |
|  |  | 5′-TCCGTTACGGACAATAAATCC-3′ |
| AtRAN3 | SALK_074683 | 5′-TTGGTGTTGAGGTTCATCCTC-3’ |
|  |  | 5′-GACAAGCCCTAACGGGTAATC-3′ |
| AtRAN4 | SALK_108184C | 5′-TCAAGAGACATCTGACTGGGG-3′ |
|  |  | 5′-GCTTCTGGTGACTCCACAAAG-3′ |
