# Supplementary material for: The Small G Protein AtRAN1 Regulates Vegetative Growth and Stress Tolerance in Arabidopsis thaliana
Source: PLoS One. 2016 Jun 3;11(6):e0154787. doi: 10.1371/journal.pone.0154787 (PMC4892486; doi:10.1371/journal.pone.0154787)
Supplement: S4 Table — (DOCX) [file pone.0154787.s008.docx]

**S4 Tab.** **Reported Ran genes Function from various plants**

| **Species** | **Genes** | **Response to stress** | **Functions** | **References** |
| --- | --- | --- | --- | --- |
| **Arabidopsis** | *Atran1* | Cold，ABA，Salt | Increase Freezing tolerance，maintenance cell division ，promote vegetative growth，increase yield | This report |
|  | *Atran2* | ND | Cell plate formation | (Ma et al. 2008) |
|  | *Atran3* | Cold，ABA | Interact with Methyl CpG bingding protein | (Yano et al. 2006) |
| **Rice** | *Osran1* | Cold | Increase cold tolerance，increase Tiller number，reduced apical dominance，maintenance cell division | ([Xu and Cai 2014](#_ENREF_45)) |
|  | *Osran2* | Cold | Increase cold tolerance，maintenance cell division | (Chen et al. 2011). |
| **Wheat** | *Taran1* | Cold | Increase cold tolerance，increase Tiller number，reduced apical dominance，maintenance cell division | (Wang et al.2006). |
| **Lepidium latifolium L.** | *LlaRan* | ND | Transgenic tobacco hypersensitive to cold | (Kim et al. 2010). |
| **Sweetpotato** | *IbRan1* | Cold，wounding | ND | (Sinha et al.2014) |
|  | *IbRan2* | Cold，osmotic |  |  |

ND; not determined.
